# Cryo-electron tomography: *en route* to the molecular anatomy of organisms and tissues

**DOI:** 10.1042/BST20240173

**Published:** 2024-12-06

**Authors:** Oda Helene Schiøtz, Sven Klumpe, Juergen M. Plitzko, Christoph J. O. Kaiser

**Affiliations:** Research Group CryoEM Technology, Max Planck Institute of Biochemistry, Martinsried, Germany

**Keywords:** cell biology, cryo-electron microscopy, FIB, lift-out, molecular ultrastructure

## Abstract

Cryo-electron tomography (cryo-ET) has become a key technique for obtaining structures of macromolecular complexes in their native environment, assessing their local organization and describing the molecular sociology of the cell. While microorganisms and adherent mammalian cells are common targets for tomography studies, appropriate sample preparation and data acquisition strategies for larger cellular assemblies such as tissues, organoids or small model organisms have only recently become sufficiently practical to allow for in-depth structural characterization of such samples *in situ*. These advances include tailored lift-out approaches using focused ion beam (FIB) milling, and improved data acquisition schemes. Consequently, cryo-ET of FIB lamellae from large volume samples can complement ultrastructural analysis with another level of information: molecular anatomy. This review highlights the recent developments towards molecular anatomy studies using cryo-ET, and briefly outlines what can be expected in the near future.

## Introduction

In the past decade, cryo-electron microscopy of single particles has elucidated a wealth of structures of macromolecules and macromolecular complexes to ever higher resolution [1]. In general, the objects of study are investigated according to a reductionist approach of purifying cellular components prior to structure determination. This, however, can be associated with side effects such as the loss of binding partners and cofactors due to a low kinetic or thermodynamic stability of the originally functional macromolecular complexes [2]. In addition, information about the spatial organization within the cells is lost due to the nature of the isolation process. One of the first comparative anatomists, Aristotle (384–322 BC) [3], albeit with a metaphysical connotation, noted that ‘[…] *all things which have several parts and in which the totality is not, as it were, a mere heap, but the whole is something beside the parts* […]’ [4]. His quote is often expressed with the words: ‘The whole is more than the sum of its parts’. Today, it is clear that the spatial and temporal organization of the parts, i.e. macromolecules, is one of the keys to the processes of life. Yet, at this point in time, while structural biology successfully studies these building blocks at great detail, the information on their cellular and supracellular level of organization has only begun to be explored structurally. It has otherwise only be inferred from complementary methods such as fluorescence microscopy.

Cryo-electron tomography (cryo-ET) is a method that can extract high-resolution structural data from biological specimens while maintaining their native, *in vivo* ultrastructure. To enable this, a key prerequisite is the preservation of the sample by cryo-fixation. As for single-particle cryo-electron microscopy, vitrification is commonly achieved through the rapid immersion of the sample in a cryogen such as liquid ethane, or a mixture of ethane and propane — a process termed plunge freezing. Vitrification of samples beyond the thickness of 5–10 µm, however, is limited by the cooling rate of the process and the sample's intrinsic heat capacity, and can result in the formation of crystalline ice [5]. Alternatively, samples up to 200 µm thick can be high-pressure frozen, a process in which a pressure of ∼2,000 bar is exerted on the sample while it is cooled in a jet of liquid nitrogen (LN_2_) [6].

Due to the technical ‘simplicity’ of preparing and processing plunge frozen samples, most cryo-ET studies have been performed on viruses, large macromolecular assemblies, unicellular organisms and cultured (adherent) cells. For transmission electron microscopy (TEM), the sample thickness should be in the range or below half the inelastic mean free path of an electron, i.e. how far an electron on average travels through a given material before it is scattered with energy loss. For biological material imaged at a beam energy of 300 kV, the inelastic mean free path is ∼300–400 nm [7]. Consequently, in order to avoid multiple scattering events, the analyzed sample area should ideally be thinner than ∼150–200 nm. Plunge-frozen samples are often sufficiently thin, or thin enough in certain regions, that they can be imaged directly with a transmission electron microscope [8–12]. Samples beyond this thickness, however, must be thinned beforehand.

Two primary methods exist to reduce sample thickness: cryo-ultramicrotomy and cryo-focused ion beam (FIB) milling. Cryo-ultramicrotomy creates a series of sections from high-pressure frozen samples by mechanical sectioning with a diamond knife [13,14]. This process is faster than FIB milling, generating multiple ultrathin sections per minute. Mastering the technique, however, requires a great amount of user expertise and talent when it comes to handling the sections. Additionally, ultramicrotomy introduces a number of artifacts such as compression and knife marks, and thicker sections, especially >70 nm, exhibit crevasses [15]. Although these artifacts may still allow for the high-resolution reconstruction of cellular macromolecular complexes (Marek Kaminek, Johannes Elferich, Wanda Kukulski, Benoît Zuber, Nikolaus Grigorieff, personal communication and [16]), correcting for the mechanical distortions at scales larger than single protein complexes remains a challenge. For this reason, cryo-FIB milling has become a more commonly deployed technique for sample thinning.

FIB microscopes, in combination with scanning electron microscopes, found broad application in the dawn of the computer era, namely for failure analysis and the modification of integrated circuits [17]. The beam, composed of ions, ablates parts of the sample very accurately (i.e. focused) to create thin sheets of material, termed “lamella”. Most commonly, Ga liquid metal ion sources and, more recently, plasma ion sources (e.g. Xe, Ar, O_2_, N_2_) are deployed. The type of ions and their properties determine the shape of the beam and the ablation rate, with plasma sources enabling faster milling at high beam currents at the expense of broader beam shapes at low currents compared with Ga sources [20,21].

More than a decade ago, FIB milling was adapted for sample preparation of frozen-hydrated/vitrified specimens [18,19]. Cryo-FIB milling has since become the primary method for plunge-frozen cellular samples for cryo-ET and, when correlated with cryogenic light microscopy data, can target specific biological events or structures [22–24]. This process is referred to as ‘on-grid’ lamella milling and, despite some adaptations, follows the original protocol: the ion beam ablates material at a shallow angle above and below a region of interest, creating a self-supporting lamella with an area of typically ∼10 × 20 µm and a homogeneous thickness of ≤200 nm [25]. This process has seen many advances in automation in recent years, and for ‘on-grid’ samples, is close to the user now only being required to load the sample, select positions and then start the run [23,26–28]. Progressing past samples that can be plunge-frozen, however, has required changes in both the vitrification and FIB milling techniques, in order to process larger volume samples in an efficient way. The decisive milestones *en route* to the molecular anatomy of whole organisms by cryo-ET, the technical intricacies and remaining challenges are the topic of the following paragraphs.

## Sample preparation for large volume specimens — getting more lamellae out of your sample

**Figure 1.**
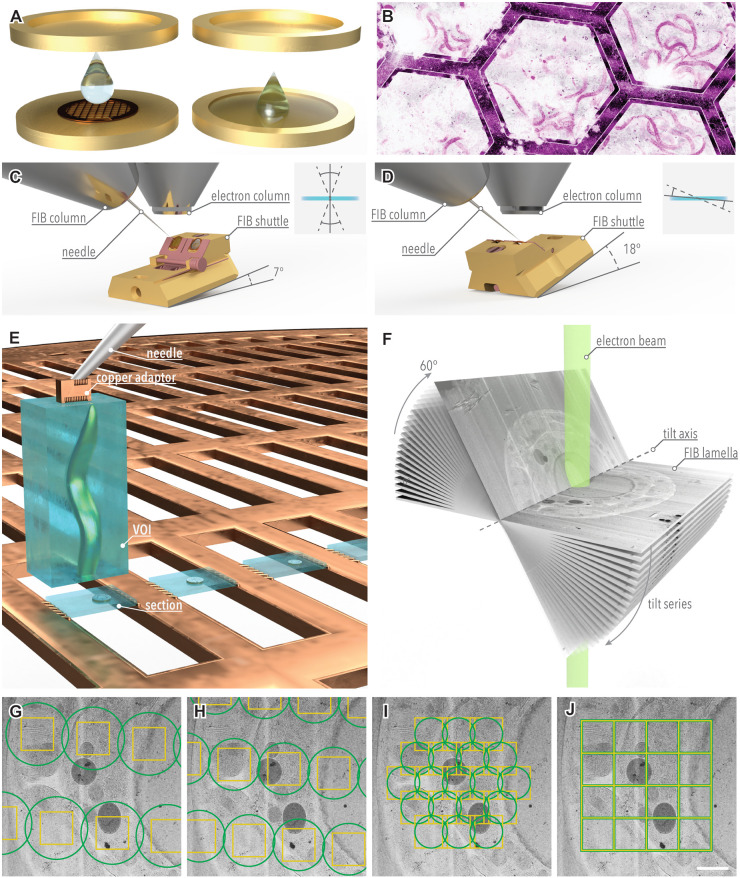
Sample preparation and data acquisition strategies for large volume cryo-ET samples. (**A–F**) Overview of the canonical workflow for cryo-ET in thick samples is shown. HPF is currently the method of choice to vitrify samples ranging from organoids, small model organisms or tissue for *in situ* cryo-ET (**A**). For ‘waffle’ samples, the specimen is applied to a TEM grid which is sandwiched in between two HPF sample carriers (left). Alternatively, the specimen is transferred into the cavity of an HPF sample carrier (right). In both cases, a cryoprotectant solution is used to allow for vitrification. Both vitrification procedures yield samples embedded within a thick ice layer. Correlative imaging is essential to identify the VOI (**B**). Here, the process is illustrated by a false-colored cryo-confocal autofluorescence micrograph of a sample of *C. elegans* L1 larvae ‘waffle’-vitrified in a hexagonal mesh EM grid. After targeting, trenches are prepared around the VOI to release it from the bulk sample material. The geometry in which the VOI is attached to the needle determines the sectioning direction against the sample surface (**C, D**). Sections (and lamellae) perpendicular to the sample plane are obtained by doing the lift-out step when the grid plane is perpendicular to the ion beam. Due to the geometry of a 45° pre-tilted shuttle, this is the case at a stage tilt of 7° (**C**). When lift-out is performed with the shuttle rotated by 180° and tilted to shallow angles (e.g. 18°), sections at shallow angles to the sample surface are obtained (**D**). The insets illustrate the sectioning plane (gray solid line) in relation to the sample surface (blue bar) as well as the range of other accessible sectioning planes for Thermo Fisher Scientific FIB instruments equipped with a cryo-rotation stage (dashed lines). Once extracted, the volume is used to prepare sections, several µm thick, by FIB-based serialized lift-out procedures as illustrated here for *C. elegans* transverse sections (**E**). These sections are thinned further to generate FIB lamellae of ideally ≤200 nm thickness in a stage position in as in **D** (not shown). The lamellae are then transferred to a TEM to acquire tilt series (**F**). Here, only one branch of a ±60° tilt series is shown for clarity on a *C. elegans* L1 larva transverse section. (**G–J**) Visualization of the closest possible lateral distribution of acquisition areas for different microscope setups and acquisition strategies. Acquisition areas (yellow) and areas illuminated by the electron beam (green) are shown on a *C. elegans* L1 larval transverse cross-section. In a standard transmission electron microscope instrument, following an equidistant acquisition setup along the tilt axis, previously exposed areas can be re-used for the low dose focus/tracking area (not shown). Due to the presence of Fresnel-fringes, generated by the out-of-focus imaging of the second condenser lens (C2) aperture edge, the exposed area has to be substantially larger than the acquisition area (**G**). While the exposed areas may slightly overlap along the tilt axis, spacing needs to be larger perpendicular to the tilt axis to avoid pre-exposure of adjacent areas caused by the elliptical distortion of the exposed area upon sample tilting. When the microscope is aligned in fringe-free imaging mode (i.e. the C2 aperture image and the sample image plane coincide on the camera), the illuminated area can be reduced to just cover the detector area (**H**). This enables closer packing of acquisition areas. Allowing for overlapping exposure areas, yet restricted to the detector area, larger coherent fields can be acquired by montage tomography in a hexagonal pattern (**I**). Note that the exposed area (green) is reduced to be smaller than the detector area (yellow). The whole pattern is rotated during acquisition to distribute the total dose evenly. A recent implementation of montage tomography harnesses a novel square geometry of apertures to optimize lateral coverage (**J**). Overlap is minimized by placing the exposed areas just next to one another. Exposure and illuminated areas are illustrated for a magnification of 42 000× on a Titan Krios G3 instrument with a Falcon 4i detector. The scale bar in panels G-J represents 1 µm.

A key step to accessing large volume samples in cryo-ET has been the application of high pressure freezing (HPF) to vitrify samples. Depending on the water content of the sample, HPF can vitrify samples up to ∼200 µm in thickness [29]. As previously stated, this is accomplished by applying ∼2,000 bar of pressure while simultaneously cooling the specimen placed inside metal carriers with a jet of LN_2_. There are currently two primary ways by which to prepare samples for cryo-ET using HPF: vitrification directly in an HPF sample carrier [30,31] or vitrification on a grid that is sandwiched between two HPF carriers (commonly referred to as ‘waffle’ freezing, Figure 1A) [32,33]. Both methods yield specimens embedded in a thick layer of ice, demanding the targeting of the biological features of interest (Figure 1B).

The ‘waffle’ method results in samples on a grid by embedding them between the grid bars in a sheet of ice ∼20–50 µm in depth, or up to ∼100 µm, if a spacer is used [32]. Alternatively, adding the sample to a grid, placing it directly into the sample carrier and filling the void cavity with 2-methylpentane produces a similar type of sample. After vitrification, the 2-methylpentane can be evaporated by heating to −170°C, so that only the exposed sample remains on the grid [34]. Both of these sample types can be used for lamella preparation directly on the grid (Figure 1A).

Samples frozen inside HPF carriers can be up to ∼200 µm thick. However, as HPF carriers are essentially metal wells, lamellae cannot be prepared directly within the carrier without additional pre-processing. Large amounts of the carrier need to either be trimmed away, exposing a sufficiently thin ledge for FIB milling, or part of the sample must first be extracted in a process referred to as lift-out (Figure 1C–E) [35–37]. As with FIB milling, this technique was adapted from materials science to cryo-ET sample preparation and utilizes either gripper-type [38] or needle-type micromanipulators [33,39–41]. Since its initial adaptation, needle-type micromanipulators have gained popularity due to their ease of use and the development of site-specific and robust attachment at cryogenic temperature. This improved attachment strategy, namely localized metal redeposition by sputtering, has replaced previous suboptimal attachment and transfer methods [42].

**Figure 2.**
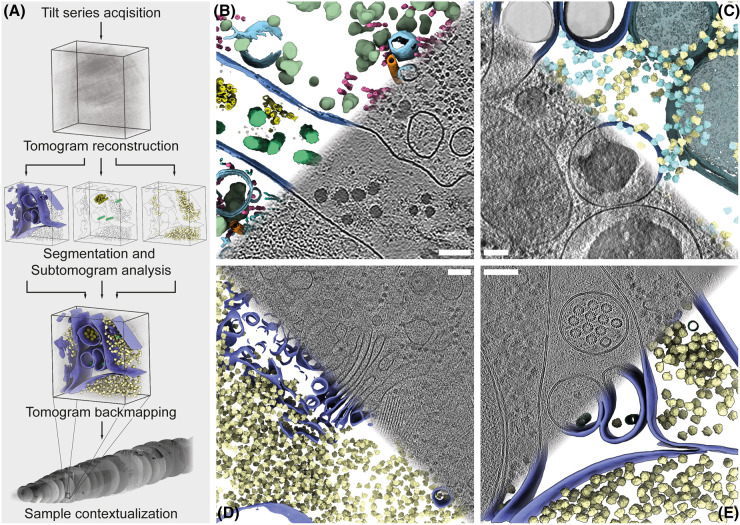
Cryo-ET of large volume samples: analysis workflow and data. (**A**) Commonly applied workflow for processing and analyzing tilt series. Acquired tilt series are first reconstructed into a tomogram. This is often followed by segmentation, particle picking, and subtomogram averaging. The results are then mapped back onto the tomogram and all of this information may then be placed back into the greater context of the sample. (**B–D**) Examples of cryo-ET analysis results from lift-out lamellae from large volume samples. Tomographic slices are shown together with their corresponding segmentations. (**B**) Cell-derived matrix of human telomerase immortalized foreskin fibroblasts. Cellular and extracellular matrix fibers are aligned perpendicular to the lamella, resulting in cross-section views. Green, collagen fibers; olive, granules; yellow, small ECM fibers; violet, plasma membrane; pink, intermediate filaments; light blue, intracellular membranes; cyan, actin filaments; beige, amorphous matrix. Adapted from [45]. (**C**) *M. musculus* islet β-cell. Cyan, pre-translocation ribosomes; yellow, post-translocation ribosomes; blue, insulin vesicles; dark green, mitochondria. Adapted from [44]. (**D**) Golgi apparatus and adjacent cellular membranes of a *D. melanogaster* egg chamber cell. Violet, membranes; yellow, ribosomes. Tomogram from the dataset acquired for [23]. (**E**) *C. elegans* L1 mechanosensititve neuron containing microtubules made up of 15 protofilaments and the cross-sections of two additional neurons containing 11 protofilament microtubules (green) in their *in vivo* organismal environment. Violet, cell membrane; yellow, 80S-ribosomes. Adapted from [47]. Scale bars: 100 nm. The ribosomes are mapped in their original locations and orientations, computationally determined by subtomogram averaging. Data in **B** kindly provided by Bettina Zens and Florian Schur. Data in **C** kindly provided by Yichun Wu and Qiang Guo.

Independent of the micromanipulator system, the overall process of lift-out remains the same: material is ablated to obtain trenches around the volume of interest (VOI), leaving only a small connection to the bulk of sample. The VOI is then gripped by or attached to the micromanipulator and released from the bulk sample by FIB milling the remaining connection. Using the micromanipulator, the VOI is then extracted and transferred to a receiver grid, where it is attached to the grid and released from the micromanipulator. The sample is then thinned down to the final lamella. Until recently, this process had high rates of failure and was very time-consuming, usually only yielding 3–4 lamellae per working day. Additionally, each individual extracted VOI would only yield a single lamella. As a collateral damage, a large amount of potentially interesting sample material was lost to ablation. This fact also holds true for lamellae generated by ‘waffle’ milling, with both techniques producing lamellae that represent <1% of the originally targeted VOI. Despite these limitations, however, the technique has been applied to assess the molecular organization of the extracellular matrix, mouse pancreatic β-cells, *Drosophila melanogaster* egg chambers and *C. elegans* (cf. Figure 2) [38,43–45].

To improve throughput and minimize the loss of contextual sample information, two techniques have recently been devised: Serial Lift-Out and Serialized On-grid Lift-In Sectioning for Tomography (SOLIST) [46,47]. For both methods, rather than extracting a small volume (e.g. 10 × 20 × 20 µm) from which a single lamella is prepared, trenches are milled around a relatively large volume (e.g. 40 × 25 × 50–200 µm). The VOI is then attached to the micromanipulator and transferred to a receiver grid where it is sectioned in an iterative fashion. The manner in which this sectioning is achieved differs between the two methods. In SOLIST, the volume is brought into the close proximity of a gold foil, spanning the receiver grid, and the sections are dropped onto the foil. The remaining volume can be moved to another mesh to repeat the process. For Serial Lift-Out, bare TEM grids are used. The volume is brought into contact with the grid bars, where redeposition milling from the bar is used to ‘weld’ the volume to the adjacent bars. A section is then released by milling through the VOI at the desired sectioning thickness (Figure 1C).

A very time-consuming step when performing single-lamella lift-out is the trench-milling step, as large amounts of material (several tens of thousands of µm^3^) have to be ablated. The time needed for the trench milling process depends on the lift-out direction and the type of sample used. Waffle grids are easier to process as no undercuts are required to release the VOI. Roughly one hour of ion beam time needs to be allocated per VOI site preparation. By limiting the amount of time spent on trench milling, i.e. a single trench for obtaining many lamellae, both recent lift-out techniques render the process of lamella generation from thick samples more time-efficient. The resulting throughput increases for e.g. Serial Lift-Out to ∼1–1.5 h/lamella in total, including thinning for a series of 20 sections.

The two techniques' approaches to sectioning do yield different advantages and drawbacks. SOLIST reports a throughput of ∼11 min per section, while for Serial Lift-Out this value is ∼16 min at 1 nA beam current. For SOLIST, however, milling a pattern of 1–2 µm is required to release a section and the minimum section thickness that was reported was 2 µm. Serial Lift-Out, while slightly slower, ensures detachment of the section from the VOI by attaching it to the receiver grid prior to sectioning. Using a line pattern to section results in only ∼500 nm of material lost to the sectioning itself, and a minimum sectioning thickness of 1 µm was achieved [46,47].

This highlights the next advantage of these sectioning techniques: the potential to obtain sequential lamellae that may not only be used to obtain high-resolution reconstructions by subtomogram averaging, but also augment contextual information for tissues and organisms by increasing the sampling density. As an example, Serial Lift-Out has been used to generate a dataset sampling the length of an entire *C. elegans* L1 larva at roughly 4 µm sectioning intervals. The larva can be approximated by a cylindrical biological volume of a diameter of 15 µm with a length of 200 µm. Assuming a final lamella thickness of 200 nm, previous approaches like ‘waffle’-milling or single region lift-out could yield roughly 6–10 lamellae, corresponding to maybe ∼350 µm^3^ of biological material. Using lift-out-based sectioning approaches this volume can be increased to ∼1800 µm^3^ by generating 50 lamellae over the same volume. This five-fold increase in available volume can be produced in about twice the time, further highlighting the increased throughput of these methods. Still, at 4 µm sectioning thickness a large fraction of the biological material is sacrificed to the lamella thinning step from the initial section thickness to ≤200 nm. Further reducing the sectioning increment to e.g. 1 µm, or below, could maintain 20% or more of the total biological volume for analysis. Notably, for a sample such as an L1 larva, when sectioning at 1 µm with a Ga-FIB, microscope time would become a major limiting factor. Sectioning alone would require multiple days and data acquisition would take close to a month, even with the most recent acquisition techniques [48].

Acceleration of the sample preparation workflow can likely be expected from using plasma ion sources for FIB milling (PFIB). Whereas the ablation rate of Xe vs. Ga at equal beam currents is roughly doubled, a tenfold higher beam current is achievable in PFIBs [49,50]. Moreover, at beam currents above ∼60 nA, the PFIB exhibits a sharper radial density distribution, increasing milling precision in the high current regime [21]. In consequence, material ablation can be achieved at ∼10-fold higher rates, removing larger volumes of sample within less time. It has also been shown that Ga-based FIB milling damages the surfaces of the lamellae by the implantation of Ga and changes to the surface chemistry [49,51,52]. Plasma ions, however, do not show implantation and have been observed to reduce the extent of the damage layer in silicon [50,53]. The assessment of plasma milling of frozen-hydrated biological material, specifically at high beam currents, has only just begun. The initial studies are encouraging and suggest that PFIB may be particularly powerful to prepare large volumes and increase the feasibility of cryo-ET studies of tissues and small organisms [49,51].

There still remains plenty of room for improvement of sectioning-based lift-out techniques, including the optimization of the fine-milling of sections, perhaps through the implementation of low energy milling schemes [54], and minimizing the material loss to the sectioning process itself. Combining serialized sectioning lift-out techniques with other complementary methods, such as integrated light microscopy and scanning electron microscopy block face imaging of the sections, is likely to yield improved targeting of regions of interest, as well as provide additional information on the material milled away during section thinning [43,55]. With automation also making great strides, including initial attempts for lift-out, it can be hoped that the techniques become available to a broader user base. The techniques expand the types of questions that can be answered, from single cell molecular sociology up to a larger scale contextual interpretation of molecular information across cells and cell types. Examples of such biological questions could be: How do known tissue-dependent differences in ribosome composition and activity translate into ribosomal molecular states and localization in a single sample? How do inclusions of neurodegenerative disease associated proteins change across cells? What structural differences are found comparing somatic cells with the germline?

## Improved data collection — getting more biology out of your lamellae

While there is still biological material lost to sectioning and the final thinning of sections, FIB milling methods now allow for a larger fraction of a VOI to be used for generating lamellae. Yet, they do not address ways by which to record the molecular information on an anatomical scale, i.e. acquire as much data as possible from the lamellae.

Recently, two major factors have contributed to improving coverage and the throughput of TEM data acquisition: being able to image areas closer to each other and being able to image multiple regions using a singular focus/tracking position. Previously, for each tilt series acquisition a nearby focus/tracking area needed to be placed along the tilt axis. To avoid prior damage to the region being imaged, these locations need to be non-overlapping along the tilt axis, and interspaced about double the beam diameter perpendicular to the tilt axis. This resulted in roughly four times the sample area recorded being allocated to and damaged by a single tilt series acquisition (Figure 1G). Additionally, the limitation of a single tilt series acquisition per focus/tracking region significantly restricted throughput. The acquisition of each tilt series following this scheme requires about half an hour, with 90% of this time spent on stage equilibration after tilting, tracking and focusing.

A key to improved coverage has been the reduction of the area damaged by electron exposure, achieved through the development of fringe-free imaging. This adjustment in the imaging system reduces the Fresnel fringes allowing the beam diameter to just include the detector area, in turn reducing the amount of sample exposed during a tilt series acquisition. Hence, acquisition areas can be located much closer to one another (Figure 1H).

Alongside this has come the adaptation of multishot data acquisition which uses beam-image shift to acquire a multitude of regions at a single stage position. Initially introduced in single particle cryo-EM, its implementation to cryo-ET was hampered by the tilting of the sample during data acquisition. This was first accomplished for plunge-frozen protein on grids by accounting for the changes in sample geometry upon tilting [56]. It reduced tilt series acquisition time to a total of ∼5 min per region acquired. To apply the technique to FIB milled samples, further adaptations were required to account for the geometry of the lamella being imaged. This has now been implemented in several software packages, including a commercially available option, Tomo5 (Thermo Fisher Scientific), and an open-source package, PACEtomo, available in the SerialEM software suite [48,57,58]. The newest version of PACEtomo, termed SPACEtomo, integrates deep learning algorithms to localize the lamellae, determine their geometry, and identify features of interest, enabling fully automated data acquisition [59].

An expansion of multishot tomography is the recent implementation of montage tomography for cryo-ET (Figure 1I–J) [60,61]. This method, not yet widely applied, uses a single focus and tracking area and records overlapping areas, covering a larger region of interest instead of imaging separate acquisition areas. This comes with challenges in optimizing positioning and overlap of the acquisition areas in order to efficiently distribute the electron dose, as well as challenges in downstream data processing. The current best practice is minimizing the beam diameter to be restricted to the detector area and acquiring in a hexagonal pattern that is shifted and rotated between tilts in a spiraling manner (Figure 1I), resulting in any given area receiving at most twice the total electron dose. The acquired tilt images can be montaged and yield a singular large field-of view tomogram, while maintaining high resolution details through the use of a small pixel size [60]. In its most recent, and still experimental implementation, square second condenser apertures are used [62,63]. The square beam formed is restricted to the detector area and thus allows acquisition areas to be placed directly beside one another with minimal overlap (Figure 1J).

While multishot and montage strategies for cryo-ET are still novel, their application shows potential to increase the amount of data that can be acquired from a sample as illustrated by recent studies on bacterial Tc toxins, nuclear basket architecture and the coronavirus double-membrane vesicle pore complex [64–66]. Especially montage tomography opens the door to perform higher resolution data acquisition while still being able to obtain a large field of view, enabling molecular anatomy studies. Analyzing the data will entail commonly applied processing such as subtomogram averaging and segmentation (Figure 2A). Expanding beyond this, however, will require new data processing pipelines to handle the ever-growing datasets and the challenges associated with their acquisition and the preservation of their spatial relations. For example, while membrane segmentation in cryo-tomograms can already be performed on larger datasets in an automated fashion [67], some analysis and annotation tasks still require skilled experts. Contextualization of the data beyond single tomograms will also require novel analysis workflows to be developed or adapted from related fields such as volume electron microscopy (Figure 2A) [68].

While the focus of this review is on the advances in TEM-based cryo-ET, it is noteworthy that great strides are also being made in scanning TEM (STEM) techniques. Recent studies illustrate that 4D-STEM, the rasterized collection of diffraction data with a pixelated detector, has great potential to contribute to the field of *in situ* structural biology [69–71]. By ptychographic reconstruction, biological macromolecules have been resolved to sub-nanometer resolution [69,71]. While not yet reaching the resolutions achieved in materials science nor leveling with single particle analyis, STEM methods applied to biological specimens have the potential to image thicker specimens (up to 1 µm) at increased contrast/detection efficiency compared with TEM. When combined with FIB-based sectioning at increments lower than 1 µm, STEM methods could consequently yield a near-complete volume of a specimen of interest, with the missing information only arising from the material lost to the sectioning process itself. This will, however, require both technological and methodological advances in FIB milling and the application of STEM to biological specimens [72].

In conclusion, although cryo-ET has been a field primarily focused on small biological samples such as viruses and single cells, recent advances in sample preparation and data acquisition are opening a door to large volume cryo-ET (Figure 2B–E). In conjunction with methods offering complementary information, such as volume electron microscopy [68], cryo-ET can add a comprehensive level of detail and completeness to answer questions about how molecular ultrastructure impacts the greater system. Consequently, Aristotle's holistic dream of understanding how the whole is more than the sum of its parts may soon become reality, albeit with a less metaphysical connotation.

## Perspectives

- *In situ* cryo-ET is an important method that enables the structural elucidation of macromolecular assemblies within their native environment.
- Recent improvements in sample processing and data acquisition techniques have made much larger volumes of specimens accessible to cryo-ET, allowing structural analysis of biomacromolecules and their molecular sociology in a close-to-native state for tissue or organismal samples <200 µm thick.
- Future automation of the sample preparation, improvements in sectioning, the ability to acquire montage tomograms, as well as the development of analysis pipelines, is likely to further increase the volume accessible to cryo-ET, rendering molecular anatomy studies technologically accessible.

## Open Access

Open access for this article was enabled by the participation of Max Planck Digital Library in an all-inclusive *Read & Publish* agreement with Portland Press and the Biochemical Society under a transformative agreement with MPDL.

## Abbreviations

cryo-ET: cryo-electron tomography
FIB: focused ion beam
HPF: high pressure freezing
STEM: scanning transmission electron microscopy
TEM: transmission electron microscopy
VOI: volume of interest
